# Interplay of N-Cadherin and matrix metalloproteinase 9 enhances human nasopharyngeal carcinoma cell invasion

**DOI:** 10.1186/s12885-016-2846-4

**Published:** 2016-10-13

**Authors:** Chih-Chin Hsu, Shiang-Fu Huang, Jong-Shyan Wang, Wing-Keung Chu, Ju-En Nien, Wei-Shan Chen, Shu-Er Chow

**Affiliations:** 1 Department of Physical Medicine and Rehabilitation, Keelung Chang Gung Memorial Hospital, Keelung, Taiwan; 2 Department of Traditional Chinese Medicine, College of Medicine, Chang Gung University, Taoyuan, Taiwan; 3 Department of Otolaryngology, Head and Neck Surgery, Chang Gung Memorial Hospital, Tao-Yuan, Taiwan; 4 Graduate Institute of Rehabilitation Science, Chang Gung University, Taoyuan, Taiwan; 5 Healthy Aging Research Center, Chang Gung University, Taoyuan, Taiwan; 6 Department of Physiology, Chang Gung University, Taoyuan, Taiwan; 7 Department of Medicine, Chang Gung University, Taoyuan, Taiwan; 8 Department of Nature Science, Center for General Studies, Chang Gung University, Taoyuan, Taiwan

**Keywords:** N-Cadherin, MMP-9, Invasion, PMA, Metastasis

## Abstract

**Background:**

N-cadherin is a trans-membrane adhesion molecule associated with advanced carcinoma progression and poor prognosis. The effect of N-cadherin on matrix metalloproteinase 9 (MMP-9) regulation is implicated in human nasopharyngeal carcinoma (NPC) cell invasion.

**Methods and results:**

Exposure of NPC cells to phorbol-12-myristate-13-acetate (PMA) or macrophage conditioned media (CM) upregulated MMP-9 and N-cadherin cleavage, which resulted in NPC cell invasion. MMP-9 cleaved the extracellular domain of N-cadherin, which was further cleaved by γ-secretase with PMA or macrophage-CM treatment. The extracellular cleavage of N-cadherin was inhibited with treatment with an MMP inhibitor and MMP-9 siRNA, whereas the intracellular cleavage of N-cadherin was inhibited by treatment with a γ-secretase inhibitor (γI), which resulted in enhanced accumulation of N-cadherin C-terminal fragment (CTF1, ~40 kDa). CTF2/N-cad (CTF2), a product of the γ-secretase cleavage of N-cadherin, was released and translocated into the nuclear compartment in PMA-treated cells. Moreover, CTF2 enhanced the effect of PMA-mediated MMP-9 gene expression as assessed by treatment with γI or overexpression with exogenous CTF2. Additionally, siRNA silencing of N-cadherin decreased PMA-mediated MMP-9 expression and cell invasion. The outside-in signaling effect of MMP-9 in macrophage CM- or PMA-treated cell cultures significantly enhanced NPC cell invasion via N-cadherin cleavage.

**Conclusion:**

Extracellular and intracellular cleavage of N-cadherin might be involved in elevated MMP-9 expression enhancing tumor cell invasion. Furthermore, N-cadherin–affected tumor progression might be via enhanced MMP-9 signaling in a cross-talk regulatory mechanism. N-cadherin might contribute to the invasive characteristics of carcinoma cells by upregulating MMP-9, thereby leading to increased aggressive metastasis.

**Electronic supplementary material:**

The online version of this article (doi:10.1186/s12885-016-2846-4) contains supplementary material, which is available to authorized users.

## Background

Human nasopharyngeal carcinoma (NPC) is a highly invasive and metastatic head and neck cancer prevalent in Southeast Asia [1, 2]. Although NPC is highly chemosensitive, chemotherapy has been associated with recurrent or metastatic NPC [3]. One of the most striking and consistent characteristics of NPC is the presence of abundant leukocyte infiltrates consisting mainly of T lymphocytes and macrophages, which suggests an important link between pro-inflammatory factors and carcinogenesis [1]. Tumor invasion is a multistep process during which cell motility is coupled with proteolysis, and this process involves cell interaction with the extracellular matrix (ECM) [4]. N-cadherin is critical for the epithelial-to-mesenchymal transition (EMT) required for highly invasive tumor growth [5]. However, the contribution of N-cadherin to carcinoma cell invasion needs investigation.

N-cadherin is a homophilic transmembrane cell adhesion molecule. Increased N-cadherin expression is a hallmark of EMT also associated with malignancy and metastasis [6]. N-cadherin promotes tumor cell survival, migration and invasion. Elevated N-cadherin level is often associated with poor prognosis [4]. Despite accumulating evidence supporting the relationship of N-cadherin level and cancer progression, the effect of N-cadherin on tumor metastasis has not been clearly demonstrated. Recent studies indicated that the key role of N-cadherin in cell adhesion and motility is its post-translational processing [5].

Metalloproteinase (MMP)-induced cadherin cleavage results in the shedding of the extracellular N-terminal amino fragment (NTF) and the generation of a first C-terminal fragment (CTF1, ~40 kDa) in the cytoplasmic compartment. CTF1 is further processed by the presenilin-1–γ-secretase complex in the juxta-membrane region, thereby releasing the cytoplasmic domain (CTF2, ~35 kDa) [4]. A regulatory function of CTFs has been implicated in cell migration and invasion [4, 7]. CTFs were recently found required for inducing MMP-9 in oral carcinoma cells [8]. MMP-9 is involved in the degradation of the ECM and cleavage of cell adhesion molecules. MMP-9 has been found to cause N-cadherin shedding that induced vascular muscle cell proliferation [9]. The study suggested that MMP-mediated proteolytic processing of N-cadherin causes shedding of its extracellular and intracellular fragments [10, 11]. The signaling properties of N-cadherininclude cross-talk with cell surface partners such as fibroblast growth factor receptors and with intracellular cascades such as the β-catenin and p120-catenin pathways [12]. Protein kinase C (PKC)–mediated ADAM10 expression has been implicated in N-cadherin cleavage leading to glioblastoma cell migration [13]. N-cadherin may enhance MMP-9 expression, thereby driving the malignant progression and invasion of tumor cells [6, 8]. MMP-9 and N-cadherin are abundantly expressed in invasive carcinoma cells [14, 15]. Thus, the dysregulation of MMP-9 and the expression of N-cadherin may be essential for promoting the aggressive invasion of carcinoma cells.

In this study, we investigated the effect of N-cadherin on MMP-9-mediated cell invasion after treatment with PMA (a potent tumor promoter) or macrophage conditioned medium (CM) in NPC cells. Upregulation of MMP-9 induced by PMA or macrophage CM stimulation mediated cell invasion via N-cadherin cleavage. Particularly, N-cadherin cleavage enhanced the expression of MMP-9. Thus, a cross-talk between N-cadherin and MMP-9 might be implicated in enhanced carcinoma cell invasion.

## Methods

### Cell culture and reagents

The human NPC cell lines NPC-TW076 and NPC-TW039 were isolated from nasopharyngeal squamous cell carcinoma [16] and maintained as previously described [2, 17]. The anti-MMP-9 antibody used for neutralizing MMP-9 activities in the conditioned medium and for western blotting was purchased from Epitomics. GM6001 (GM), a broad-spectrum MMP inhibitor, MMP9I, a potent, selective and reversible MMP-9 inhibitor, and L-685,458 (γI), an inhibitor of N-cadherin cleavage were from BioVision. A mouse anti-N-cadherin antibody (610920, clone 32, BD Biosciences) was used to recognize the intracellular domain of N-cadherin. Other antibodies were from Cell Signaling. Additional inhibitors, U0126 (MEK1/2 inhibitor), SB203680 (p38 mitogen-activated protein kinase [MAPK] inhibitor), SP600125 (JNK inhibitor), and bisindolylmaleimide (BIM, a protein kinase inhibitor), were from BioVision or Enzo Life. 6-Amino-4-(4-phenoxyphenylethylamino) quinazoline (QNZ), a NFκB activation inhibitor, was from Cayman International.

### Collection of conditioned media

NPC cells were seeded in 6-well plates at 3 × 10^5^ cells per well. Cells were cultured with and without PMA (100 nM) for 10 h, washed with 1 X phosphate-buffered saline (PBS) three times to completely remove PMA, then incubated with fresh medium for 24 h. The resulting PMA-treated and -untreated conditioned media (CM1 and C1, respectively) were collected and stored at −20 °C. THP1-derived macrophages were generated as described [18]. THP-1 monocytes were seeded in 6-well plates at 4 × 10^6^ cells per well in 3 ml completed medium briefly. Cells were treated with 100 nM PMA for 24 h, washed three times with 1X PBS, then incubated for 48 h in 2 ml fresh completed medium. The resulting macrophage CM (mϕCM) was collected, clarified by centrifugation, then stored at −20°C. Conditioned media from THP-1 monocytes (monoCM) was harvested similarly.

### siRNA transfection

Specific small interfering RNAs (siRNAs) were used to silence MMP-9 and N-cadherin expression. An siRNA targeting part of the N-cadherin mRNA was selected and synthesized by Pharmacon Research Inc. The MMP-9-specific siRNA was from Santa Cruz Biotechnology and the negative control siRNA (Ngi), a scramble, was as previously described [19]. NPC cells were transfected with siRNA duplexes according to the manufacturer’s protocol.

### Preparations of cell lysates, subcellular fractions and western blot analysis

Before exposure to PMA or mϕCM, cells were seeded in 6-well culture plates at 3 × 10^5^ cells/well and treated with siRNA or the inhibitors BIM (2 μM), γI (5 μM) or MMP-9I (20 μM), U0126 (20 μM), SB203580 (20 μM), SP600125 (20 μM) or QNZ (20 μM) [20, 21]. Cytosolic and nuclear extracts were prepared by using the NE- PER nuclear and cytoplasmic extraction reagents kit (Thermo Fisher Scientific). The cellular and subcellular extracts were prepared and separated on SDS-PAGE and blotted onto polyvinylidene difluoride membranes (Immobilon (TM)-P, Millipore) as described [17]. Blots were probed with primary antibodies then appropriate horseradish peroxidase-conjugated secondary antibodies. Immunoreactive protein bands were developed with the Enhanced Chemiluminescence reagent (Perkin Elmer LAS Inc.).

### Matrigel-coated invasion assay

The Boyden chamber invasion assay involved use of 6-mm Transwell chambers containing polycarbonate membranes with 8-μm pores (Becton-Dickinson). Cells were re-suspended in medium containing 1 % fetal bovine serum (FBS) and added to Matrigel-coated upper chambers at 4 × 10^4^ to 1 × 10^5^ cells per well. The lower chamber was filled with DMEM containing 5–10 % FBS with and without 100 nM PMA or CM. After 24 h, cells that migrated to the lower wells were fixed in 4 % paraformaldehyde, stained with 0.25 % crystal violet, and counted under a light microscope in five predetermined fields (100X or 200X).

### Gelatin zymography assay

CM was separated by 10 % SDS-PAGE with gels containing 0.1 % gelatin. After electrophoresis, gels were washed twice in washing buffer (2.5 % Triton X-100 in dH_2_O) at room temperature for 30 min each time to remove SDS, then incubated in reaction buffer (10 mM CaCl_2_ and 40 mM Tris–HCl, pH 8.0) at 37 °C for 12 h to allow proteolysis of the gelatin substrate. The bands corresponding to the expression of MMP-9 were visualized by negative staining with Coomassie Brilliant blue R-250 (Bio-Rad Laboratories), and molecular weights were estimated by referencing pre-stainedSDS-PAGE markers.

### Construction of pMMP-9-Luc and pEGFP-CTF2 plasmids and promoter reporter assay

An MMP-9 promoter fragment spanning nucleotides −925 to +13 was synthesized from human genomic DNA (Promega) by PCR as described [22]. The amplified PCR products were ligated into the pGL3-basic vector (Promega) to generate the plasmid pMMP9-Luc. Transient transfection of the luciferase reporter plasmids involved use of Lipofectamine 2000 (Invitrogen) according to the manufacturer’s instructions. NPC cells were seeded in 12-well plates and transfected with 0.5 μg pMMP9-Luc and 0.5 μg pSV-β-galactosidase control vector (Promega). At 24 h post-transfection, the levels of firefly luciferase activity were measured in each sample by using the Luciferase Assay System (Promega). The β-galactosidase enzyme assay involved use of the same lysates to standardize the transcription efficiency. The relative amount of luciferase activity in the untreated cells was set to 1.

Human N-cadherin in pCCL-c-MNDU3c-PGK (pCCL-c-MNDU3c-PGK-EGFP) was a gift from Nora Heisterkamp (Addgene plasmid #38153) [23]. The cytoplasmic fragment (CTF2) of N-cadherin cDNA (Accession no. NM_001792, 2663–3145 bp) was amplified by PCR with the primer sequences 5′-CGAGCTCAAGCTTCGAAAC GCCGGGATAAAGAACG-3′ and 5′-TACCGTCGACTG CAGTCAGTCATCACCTCCACCAT-3′. The pEGFP-CTF2 plasmid was assembled from the CTF2 cDNA and the EcoRI-linearlized plasmid pEGFP-C1 (Clonetech, CA). The DNA recombination involved use of the GeneArt Seamless Cloning and Assembly Kit (Invitrogen).

### Activation of pro-MMP-9 in vitro

Pro-MMP-9 (R&D Systems, 911-MPN-010) was activated by incubation for 24 h at 37 °C in TCNB buffer (50 mM Tris, 10 mM CaCl_2_, 0.15 M NaCl, and 0.05 % Brij). NPC cells were incubated with activated MMP-9 (0.1 nM) for 7 h, and the effects of MMP-9 on cellular N-cadherin and cell invasion were determined.

### Statistical analyses

Data are presented as mean ± SEM. The Kruskal–Wallis test was used to compare differences in protein levels among the three cell lines. Differences between any two proteins were estimated by Dunn’s multiple comparisons test. The Mann–Whitney U test was used to assess the pre- and post-treatment protein levels in each cell line. *P* < 0.05 was considered statistically significant.

## Results

### N-cadherin cleavage mediated the m ϕ CM-increased expression of MMP-9

**Fig. 1:**
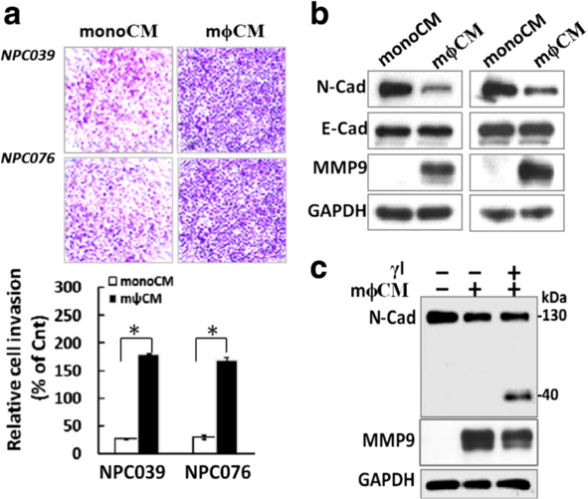
Blockade of N-cadherin cleavage decreased m ϕ CM-induced cell invasion and MMP-9 levels. **a** m ϕ CM enhanced NPC cell invasion. NPC cells were seeded into the inner well of the Boyden chamber, pre-coated with matrix-gel and monoCM or m ϕ CM, then introduced into the outer well of the Boyden chamber for 24 h. Cells that invaded the lower surface of the filter were fixed, stained, photographed and counted. Representative plots of Matrigel invasion assay are shown. Data are mean±SEM. *N* = 3, ^*^ *P*<0.05. **b** NPC cells were treated with monoCM or m ϕ CM for 24 h, then cell lysates underwent western blot analysis with indicated antibodies (N-cad, E-cad and MMP-9). **c** Blockade of N-cadherin cleavage by γI decreased m ϕ CM-induced MMP-9 expression in NPC-TW039 cells. NPC cells were pre-treated with γI (5 μM), then exposed to m ϕ CM for 24 h. Cell lysates underwent western blot analysis with indicated antibodies

Tumor-associated macrophages are commonly found at the invasive fronts of advanced carcinoma [24]. We investigated the involvement of N-cadherin in macrophage-induced NPC cell invasion. Cell invasion was greater in mϕCM- than monoCM-treated NPC cells (Fig. 1a). Furthermore, exposure to mϕCM increased MMP-9expression and decreased the expression of full-lengthN-cadherin (FL/N-cad, ~130 kDa) but did not affect E-cadherin expression (Fig. 1b). L-685,458, a γ-secretaseinhibitor (γI), can prevent the intracellular processing of the C-terminal fragments of N-cadherin (CTFs/N-cad) [25]. mϕCM affected the intracellular cleavage of N-cadherin as seen by increased CTF1 expression and decreased MMP-9 expression with γI treatment (Fig. 1c). Thus, mϕCM enhanced the intracellular cleavage associated with mϕCM-increasedMMP-9 expression.

#### MMP-9 induced N-cadherin cleavage and cell invasion after PMA treatment

**Fig. 2:**
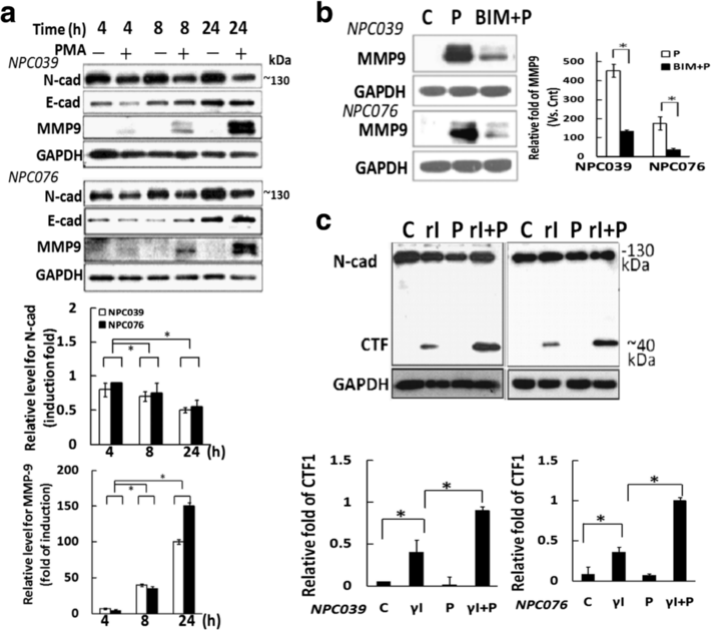
PMA induced the cleavage of N-cadherin.**a**N-cad and MMP9 expression with/without PMA treatment. NPC cells were exposed to PMA at the indicated times, and cell lysates underwent immunoblotting. The relative band intensities of N-cadherin and MMP-9 proteins were normalized to GAPDH by densitometry. Data are mean±SEM. *P<0.05, *N* = 3. **b** PMA induced MMP-9 expression via a PKC-dependent pathway. NPC cells were pre-treated with BIM (2 μM) for 2 h, then PMA (100 nM) for 24 h. Cell lysates underwent western blot analysis with the indicated antibodies. **c** PMA induced the cleavage of N-cadherin. NPC cells were pre-treated with L-685,458 (5 μM, γI), then exposed to PMA for 24 h. Cell lysates underwent western blot analysis with the indicated antibodies. The ratio of FL/N-cad or CTF/N-cad to GAPDH was calculated. Data are mean±SEM. *N* = 3, ^*^ *P*<0.05

PKCs are involved in the initiation of N-cadherin cleavage and cell invasion in glioblastoma cells [13]. To examine the involvement of N-cadherin in NPC cell invasion, NPC cells were treated with PMA for various times. PMA time-dependently upregulated MMP-9 and downregulated FL/N-cad but did not affect the expression of E-cadherin (Fig. 2a). The observed PMA-inducedMMP-9 upregulation was blocked by 1-hpre-treatment with the broad-spectrum PKC inhibitor bisindolylmaleimide (BIM) (Fig. 2b), so the process was PKC-inducible.

CTF1/N-Cad (CTF1) is the product of extracellular metalloproteases that cleave close to the interface between the extracellular and transmembrane regions of N-cadherin [10]. CTF2/N-Cad (CTF2) results from subsequent γ--secretase cleavage, which follows the removal of the extracellular region by extracellular MMPs [26]. To analyze the possible PMA-mediatedN-cadherin intracellular cleavage, the amount of CTF1 was validated in NPC cells pre-treated with γI, then co-incubated with and without PMA for 24 h. The intracellular CTFs were recognized by the mouse anti-N-cadherin antibody (BD Bioscience). The baseline accumulation of CTF1 (~40 kDa) was detected in cells with γI treatment (Fig. 2c). Remarkably, PMA treatment after γI treatment increased CTF1 expression approximately 2.3- and 3.1-fold in NPC-TW039 and NPC-TW076 cells, respectively (Fig. 2c). Therefore, PMA upregulated MMP-9 and the extracellular and intracellular cleavage of N-cadherin.

### N-cadherin cleavage enhanced MMP-9 expression

**Fig. 3:**
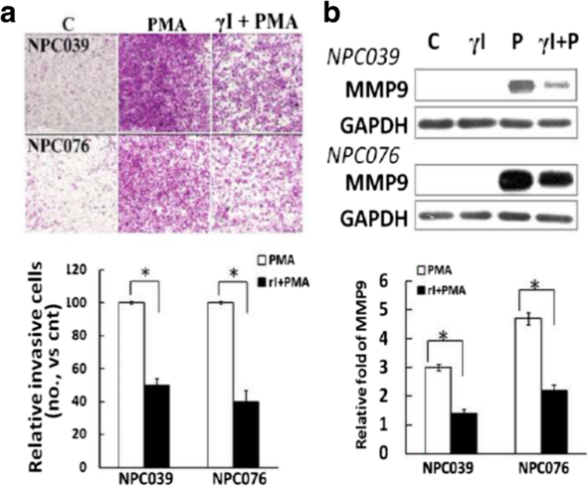
Blockade of N-cadherin cleavage decreased PMA-mediated cell invasion and MMP-9 level. **a** γI treatment decreased PMA-induced cell invasion. NPC cells were treated with γI in the inner well of the Boyden chamber, and PMA (100 nM) plus γI was introduced into the outer well for 24 h. Cells that invaded the lower surface of the filter were fixed, stained and photographed. Representative images and statistical plots of the invasion assay are shown. The relative cell invasion in the γI-plus-PMA–treated group was compared to PMA treatment alone. **b** γI treatment decreased the PMA-mediatedMMP-9 expression. NPC cells were pre-treated with γI (5 μM), then exposed to PMA for 24 h. Cell lysates underwent immunoblotting with the indicated antibodies. The relative MMP-9 expression was compared with the control group (PMA-untreated cells); *N* = 3, ^*^ *P*<0.05

We investigated the role of N-cadherin cleavage in NPC cell invasion. PMA-mediated cell invasion was markedly abrogated by treatment with γI (Fig. 3a). Importantly, γI treatment markedly reduced PMA-upregulatedMMP-9 and increased PMA-mediated accumulation of CTFs/N-cad (Figs. 3b and 2c). Thus, cleavage of N-cadherin was implicated in upregulated MMP-9 expression.

### MMP-9 induced N-cadherin cleavage

**Fig. 4:**
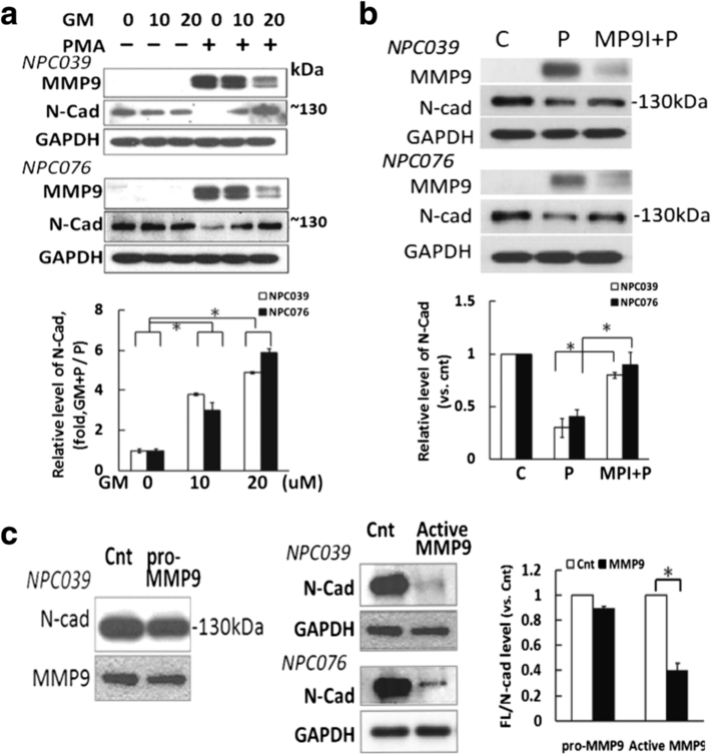
MMP-9 inhibitors abolished PMA-inducedN-cadherin cleavage. NPC cells were pre-treated with (**a**) GM6001 (GM, 10–20 μM) or (**b**) a specific MMP-9 inhibitor (20 μM) for 2 h, then co-incubated with PMA (100 nM) for 24 h. Cell lysates underwent immunoblotting with the indicated antibodies. The relative band intensities of FL/N-cad were compared with the control group (PMA-untreated cells), and relative expression (RLE) of N-cadherin is shown as the ratio of GM-plus-PMA–treated group to PMA treatment alone. **c** The effect of pro-MMP-9 or activated MMP-9 on the cleavage of N-cadherin.Pro-MMP-9 (R&D, 911-MPN-010) was activated by incubation in TCNB buffer for 24 h at 37 °C. NPC-TW039 cells were exposed to activated MMP-9 or pro-MMP-9 (0.1 nM) for 7 h. Cell lysates underwent western blot analysis with anti-N-cadherin antibody. The relative expression of FL/N-cad was compared to the control group (activated MMP-9-untreated cells); *N* = 3, ^*^ *P*<0.05

We investigated the role of MMP-9 in N-cadherin cleavage by treatment with an MMP inhibitor. PMA-upregulatedMMP-9 expression was effectively abrogated by treatment with a broad-spectrum MMP inhibitor (GM6001, GM) that markedly diminished the cleavage of N-cadherin (Fig. 4a), and the PMA-mediated cleavage of N-cadherin and MMP-9 was markedly inhibited by a specific MMP-9 inhibitor (MP9I, Fig. 4b). To further validate the function of MMP-9 in N-cadherin cleavage, NPC cells were exposed to pure, activated or inactivated recombinant MMP-9 protein. Treatment with pro-MMP-9 slightly decreased the expression of N-cadherin, but exposure to activated MMP-9 for 7 h significantly reduced the expression of FL/N-cad (Fig. 4c). Hence, activated MMP-9 induced the extracellular cleavage of N-cadherin. However, the extracellular cleavage of N-cadherin on treatment with activated recombinant MMP-9 alone did not induce NPC cell invasion (data not shown).

### PMA induced MMP-9 expression via activation of MAPK or NF-κB signaling

**Fig. 5:**
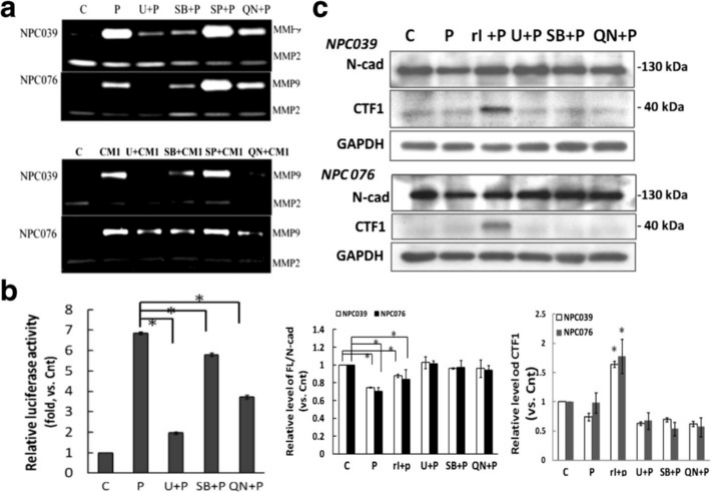
PMA or CM1 upregulated MMP-9 via the ERK, p38 MAPK and NF-κB pathways. **a** PMA (m ϕ CM) induced MMP-9 expression via activation of MAPK and NF-κB pathways. NPC cells were pre-treated with specific inhibitors of ERK 1/2 (U0126, 10 μM), p38 (SB203580, 10 μM), JNK (SP600125, 10 μM) or NF-kB (QNZ, 10 μM) for 1 h, then treated with PMA (100 nM) or m ϕ CM for 18 h, and MMP-9 levels in the CM were determined by gelatin zymography. The relative level of MMP-9 expression was compared in cells treated with MAPK or NF-κB inhibitor plus PMA and PMA treatment alone; *N* = 3, ^*^ *P*<0.05. **b** PMA induced the reporter activity of MMP-9 in NPC cells. NPC 039 cells were transfected with pGL-MMP9-Luc for 24 h. Transfected cells were treated with U0126, SB203580, or QNZ for 1 h, then exposed to PMA (100 nM) for 6 h. Luciferase activity is presented relative to the control group. **c** MAPK or NF-kB signaling was not directly involved in the regulation of the intracellular cleavage of N-cadherin. NPC cells were pretreated with the indicated inhibitors for 1 h before 8 h of PMA exposure. Cell lysates underwent immunoblotting with the indicated antibodies. The relative CTF1 accumulation was compared with the control group (PMA-untreated cells); *N* = 3, ^*^ *P*<0.05

During tumor progression, multiple signaling pathways control the expression of PMA-mediatedMMP-9 [27]. We investigated the involvement of MAPKs (extracellular signal-regulated kinase 1/2 [ERK1/2], p38, and c-JunN-terminal kinase [JNK]) and NF-κB signaling during PMA-inducedMMP-9 expression and N-cadherin cleavage. Treatment with inhibitors of ERK1/2 and p38 or NF-κB abrogated the PMA-upregulatedMMP-9 expression as investigated by gelatin zymography (Fig. 5a). To examine the effect of the MAPKs or NF-κB signaling on the reporter activity of MMP-9, NPC cells were transfected with pGL-MMP9-Luc for 24 h and treated with an MAPK or NF-κB inhibitor before exposure to PMA. Luciferase activity induced by PMA was significantly suppressed on treatment with an ERK1/2, p38 or NF-κB inhibitor (Fig. 5b). Next, we examined the effect of MAPK or NF-κB signaling on cleavage of N-cadherin.PMA-treated NPC cells with an ERK1/2, p38 or NF-κB inhibitor incubation showed no accumulation of CTF1/N-cad induced by γI (Fig. 5c). Conversely, the inhibitors markedly abolished the PMA-mediated cleavage of FL/N-cad. PMA-mediatedMMP-9 expression may be via activation of ERK1/2, p38 or NF-κB signaling. However, MAPK or NF-κB signaling did not directly mediate the intracellular cleavage of N-cadherin.

### Cross-talk between N-cadherin and PMA-mediatedMMP-9

**Fig. 6:**
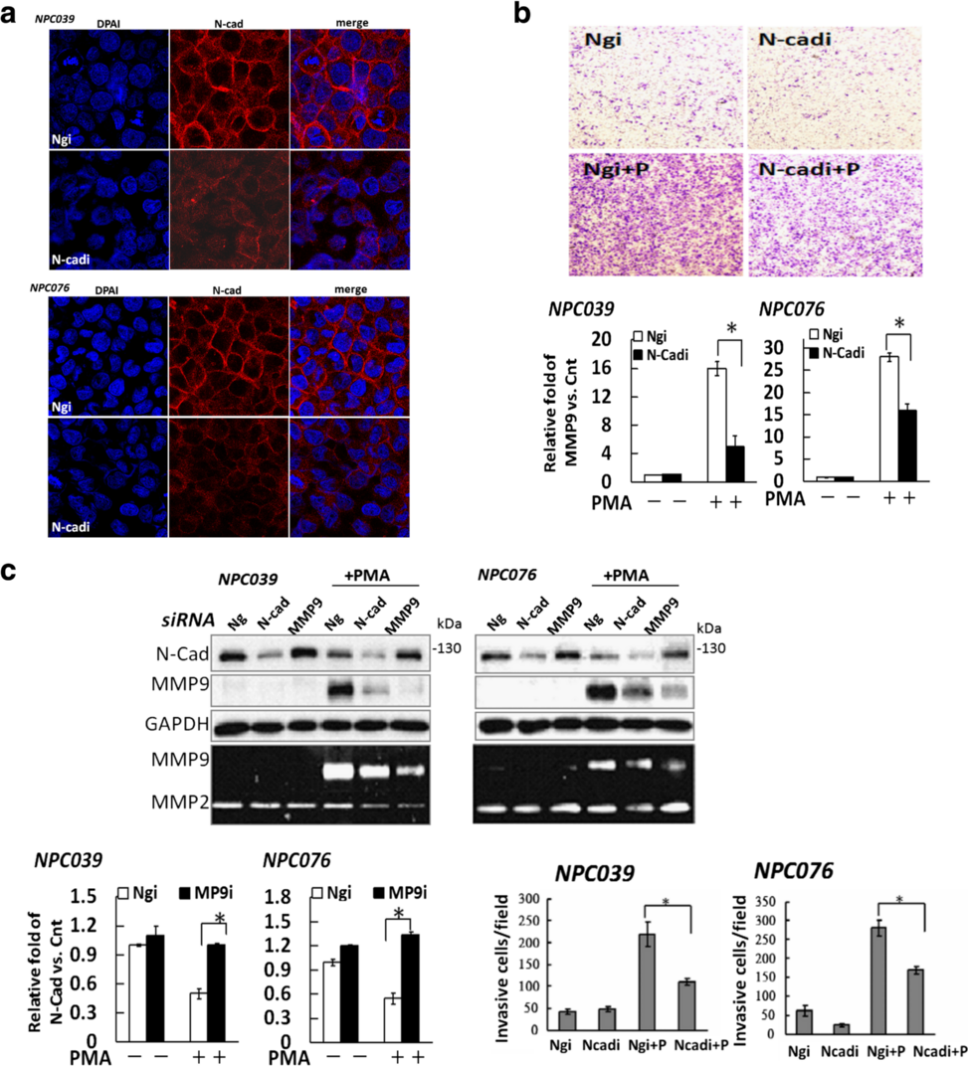
Cross-talk regulation between N-cadherin cleavage and MMP-9 expression. **a** SiRNA silencing of N-cadherin disrupted the cell–cell adhesion. NPC cells were transfected with N-cadherin siRNA for 24 h, then examined by immunofluoresence staining with anti-N-cadherin antibodies and analyzed by confocal laser scanning microscopy. **b** Silencing of N-cadherin decreased PMA-mediated cell invasion. NPC cells were transfected with N-cadherin siRNA for 24 h, then cell invasion was investigated by Boyden chamber assay. Representative images are shown and data are presented as mean±SEM. **c** NPC cells were transfected with siRNA specific for MMP-9 or N-cadherin for 24 h, then exposed to PMA for 24 h. The transfected cells were cultured with and without PMA for 24 h. The expression of MMP-9 and N-cadherin in cell lysates was assessed by western blot analysis with the indicated antibodies. MMP-9 level in CM was detected by gelatin zymography. The relative expression of FL/N-cad or MMP-9 in MMP-9 siRNA- or N-cadsiRNA-transfected cells was compared with Ng (non-specific control) siRNA (Ngi) -transfected cells after PMA treatment; *N* = 3, ^*^ *P*<0.05

We examined the contribution of N-cadherin in NPC cells and evaluated the cell–cell adhesion phenotype of NPC cell lines that expressed N-cadherin by immunofluoresence staining with N-cadherin antibodies. Control cells with higher intensities of N-cadherin appeared similar to cobblestones (Fig. 6a), whereas N-cadherinsiRNA-transfected cells seemed scattered and may be lost in cell–cell contact. To examine the role of N-cadherin in cell invasion, NPC cells were transfected with N-cadherin siRNA for 24 h, then exposed to PMA. The invasive capacity was lower for of N-cadherinsiRNA-transfected cells than negative control siRNA (Ngi)-transfected cells (Fig. 6b). To independently validate the interaction between N-cadherin and MMP-9, NPC cells were transfected with MMP-9 or N-cadherin siRNA for 24 h before PMA treatment. SiRNA knockdown of MMP-9 efficiently reduced the expression of MMP-9 and significantly abolished the PMA-mediated cleavage of N-cadherin (Fig. 6c). SiRNA silencing of N-cadherin significantly reduced PMA-upregulatedMMP-9 expression. MMP-9 in CM consistently exhibited a similar effect, as investigated by gelatin zymography (Fig. 6c). The data suggest a cross-talk between N-cadherin and MMP-9 expression in modulating PMA-mediated carcinoma cell invasion.

### Upregulation of MMP-9 via N-cadherin cleavage

**Fig. 7:**
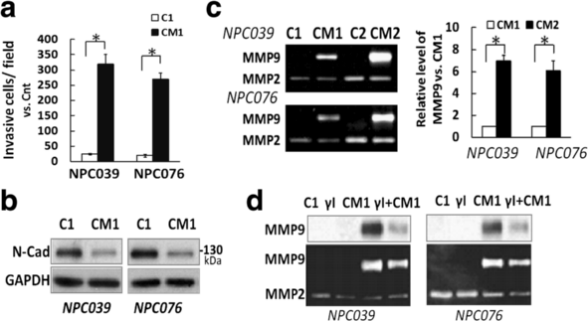
CM1 significantly enhanced cell invasion accompanied by increments of MMP-9 upregulation and N-cadherin cleavage. Briefly, NPC cells were cultured with and without PMA for 8 h, then PMA-containing medium was completely removed and replaced with completed medium for 24 h. The CM was collected and referred to as CM1 or C1. **a** CM1 induced NPC cell invasion. The invasive capability of CM1-treated NPC cells was investigated. CM1 was introduced into the outer well of NPC-cell–seeded Boyden chambers for 24 h. The invasive cells at the lower surface of the membrane were fixed, stained, photographed and counted. Data are mean±SEM. *p<0.05, *N* = 3. **b** CM1 reduced the level of N-cad. NPC cells were exposed to CM1 or C1 for 24 h, then cell lysates underwent western blot analysis. **c** CM1 upregulated MMP-9. NPC cells were treated with CM1 or C1 for 24 h, the CM was collected and referred to as CM2 or C2, respectively. The level of MMP-9 in CM1/CM2 was examined by gelatin zymography. Data are mean±SEM. *N* = 3, ^*^ *P*<0.05. **d** Blockade of intracellular cleavage of N-cadherin decreased CM1-mediatedMMP-9 expression. NPC cells were pre-treated with γI for 2 h and co-incubated with CM1 for 24 h, then cell lysates and CM underwent western blot analysis and gelatin zymography

The possible outside-in signaling of MMP-9 via N-cadherin cleavage warrants further exploration. NPC cells were cultured with and without PMA for 8 h, washed with 1X PBS three times then incubated with normal culture medium for 24 h. The conditioned media from the PMA-treated and untreated NPC cells was collected (CM1 and C1, respectively). NPC cells were exposed to CM1 or C1 for 24 h, and the effect of CM1 or C1 on invasion was analyzed by matrix-gel–coated transwell assay. CM1 incubation significantly increased NPC cell invasion (Fig. 7a) and reduced FL/N-cad protein level (Fig. 7b). The harvested CM of CM1- and C1-treated cells was referred to as CM2 or C2, respectively, and the level of MMP-9 in CM1 and CM2 was compared on gelatin zymography. MMP-9 expression was markedly greater in CM2 than CM1 (Fig. 7c). Remarkably, CM1-mediatedMMP-9 expression in cell lysates and CM was abrogated on γI treatment (Fig. 7d). The data also suggest that intracellular cleavage after CM1 treatment plays an important role in increased MMP-9 expression.

**Fig. 8:**
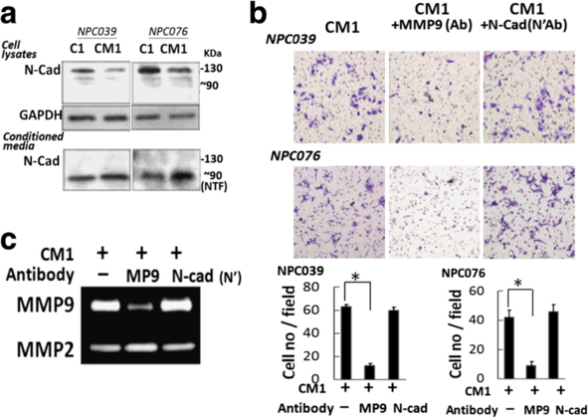
CM1-derived MMP-9 mediated cell invasion. C1 or CM1 was harvested as described in Fig. 7. **a** NTF/N-cad was detected in CM1. Equal amounts of cell lysate or CM underwent western blot analysis with an anti-N-cadherin antibody (sc-7939) that recognizes the extracellular domain (FL/N-cad, ~130 kDa; NTF/N-cad, ~90 kDa). **b **Co-incubation with an anti-MMP-9 antibody decreased CM1-mediated cell invasion. CM1 was co-incubated with antibodies for IgG, MMP-9 or NTF/N-cad for 2 h, then introduced into the outer well of the Boyden chamber for 24 h. Cells on the lower surface of the membrane were fixed and stained with crystal violet. Data are mean±SEM. *N* = 3, ^*^ *P*<0.05. **c** The expression of MMP-9 in the CM from the outer well of the Boyden chamber was determined by gelatin zymography

The NTF (~90 kDa) of N-cadherin plays a role in modulating cell invasion [28]. PMA treatment might induce the expression of various signaling molecules (including the NTF) and their release into the CM. Cell lysates or CM1 underwent western blot analysis with an anti-NTF/N-cad antibody that recognizes the N-terminal fragment of N-cadherin. A soluble fragment of ~90 kDa was detected in CM1 but not in cell lysates (Fig. 8a ). We investigated the invasive capacity of NTF/N-cad and MMP-9 in CM1. CM1 was pre-incubated with the antibodies for IgG, MMP-9 or NTF/N-cad for 2 h before exposure to NPC cells for 24 h, then cell invasion and MMP-9 levels in CM were observed. CM1 pre-incubated with anti-MMP-9 antibody did not trigger NPC cell invasion (Fig. 8b) and was accompanied by reduced levels of MMP-9 (Fig. 8c). Therefore, MMP-9 in the CM1 mediated NPC cell invasion.

### PMA increased the CTF nuclear translocation and the reporter activity of MMP-9

**Fig. 9:**
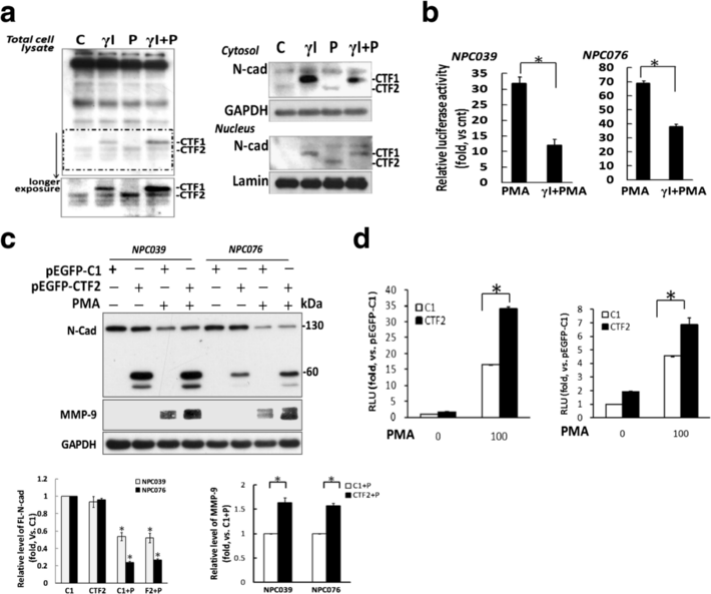
CTF2 enhanced the PMA-mediatedMMP-9 gene expression. **a** The nuclear translocation of CTFs/N-Cad was blocked by pretreatment with γI before exposure to PMA. NPC cells were pretreated with γI for 2 h, then co-incubated with PMA for 16 h. Total cell lysates and sub-cellular fractions underwent western blot analysis, and levels of CTFs/N-Cad were detected. GAPDH was a loading control for the cytosol fraction. Lamin B1 was a loading control for the nuclear fraction. **b** The reporter activity of PMA-mediatedMMP-9 was inhibited after blockade of N-cadherin intracellular cleavage. NPC cells were transfected with pGL-MMP9-Luc for 24 h, then pre-treated with γI for 2 h and co-incubated with PMA for 16 h. β-galactosidase activity in each transfection was used to normalize luciferase activity. The luciferase activity is presented relative to the control group (PMA-untreated cells). Data are mean±SEM. *N* = 3, ^*^ *p*<0.05. **c** Exogenous expression of CTF2/N-cad enhanced PMA-upregulatedMMP-9 expression. NPC cells were transfected with pEGFP-C1 (C1) or pEGFP-CTF2 (CTF2), then treated with PMA for 16 h. Cell lysates underwent western blot analysis with the indicated antibodies. Exogenous CTF2-conjugated EGFP is shown as a band of ~60 kDa. Data are mean±SEM. *N* = 3, ^*^ *P*<0.05. **d** Exogenous expression of CTF2 enhanced the reporter activity of MMP-9. NPC cells were transfected with p-EGFP-CTF2 or pEGFP-C1 in the presence of pGL-MMP9-Luc for 24 h, then exposed to PMA for 6 h. Luciferase activity is relative to the pEGFP-C1- transfected group. Data are mean±SEM. *N* = 3, ^*^ *P*<0.05

We assessed the possible signaling of CTFs of N-cadherin in regulating MMP-9. NPC cells were pre-treated with γI, then co-incubated with PMA for 8 h; the distribution of CTFs in total cell lysates and cytosol/nuclear fractions was detected. Consistent with results in Fig. 3, treatment with γI increased CTF1 level in total cell lysates distributed in the cytsol and nuclear fractions (Fig. 9a, lane 2 in left and right panels, respectively). We then investigated the subcellular distribution of CTF2. Treatment with PMA increased CTF2 level in total cell lysates distributed in the cytosol and nuclear fractions (Fig. 9a, lane 3 in left and right panels, respectively). Treatment with γI abrogated this PMA-mediated CTF1 cleavage, which resulted in enhanced accumulation of CTF1 and barely detectable amounts of CTF2 in total cell lysates and in cytosol or nuclear fractions (Fig. 9a, lane 4 in left and right panels, respectively). Thus, PMA-induced CTF1 cleavage may result in nuclear localization of CTF2.

Next, we transfected NPC cells with pGL-MMP9-Luc for 24 h, then transfected cells were pretreated with γI and co-incubated with PMA for 16 h. γI significantly inhibited the luciferase activity induced by PMA, which implies a signaling effect of intracellular cleavage of N-cadherin on upregulated MMP-9 (Fig. 9b).

To determine whether CTF2 upregulated MMP-9, we constructed a pEGFP-CTF2 plasmid with CTF2/N-cad and EGFP. NPC cells were transfected with pEGFP-CTF2 or pEGFP-C1 (empty vector with EGFP) along with pGL-MMP9-Luc for 24 h and examined for PMA-inducedMMP-9 expression in NPC cells expressing an exogenous CTF2 domain (with EGFP) (~60 kDa) (Fig. 9c). Overexpression of CTF2 significantly enhanced the PMA-upregulatedMMP-9 protein level. To examine the effect of CTF2 on the reporter activity of MMP-9, NPC cells were co-transfected with pEGFP-CTF2 or pEGFP-C1 along with pGL-MMP9-Luc for 24 h before exposure to PMA for 6 h. Luciferase activity was significantly enhanced with CTF2 overexpression (Fig. 9d). Thus, CTF2 could enhance the effect of PMA on MMP-9 gene regulation.

## Discussion

N-cadherin, a hallmark of EMT, is associated with carcinoma cell metastasis. This study demonstrated novel roles for N-cadherin and MMP-9 in the NPC metastasis process. Shedding of N-cadherin may contribute to the invasion of carcinoma cells via upregulation of MMP-9. Exposure to PMA or macrophage CM induced post-translational cleavage of N-cadherin. Disruption of MMP-9- or γ-secretase–mediated N-cadherin cleavage downregulated MMP-9 and linked these processes to carcinoma invasion. Overexpression of CTF2/N-cad enhanced the PMA regulation of MMP-9 expression. The outside-in signaling effect of macrophage CM mediated by N-cadherin cleavage depended on γ-secretase. We identified a cross-talk between MMP-9 and N-cadherin cleavage involved in the regulation of carcinoma cell invasion, which provides a possible mechanism for MMP-9 in inflammatory-mediated cell invasion via N-cadherin cleavage.

Inflammation is a driving force in carcinoma cell metastasis [24]. A high density of infiltrated inflammatory cells, particularly tumor-associated macrophages, is commonly found at the invasive front of advanced carcinoma [24]. The infiltrated cells secrete a wide variety of growth factors and cytokines to stimulate the growth, motility and invasiveness of tumor cells. MMPs mediate the enhanced invasive ability of tumor cells co-cultured with macrophages or macrophage CM [24]. Here, we investigated the outside-in signaling of MMP-9 in NPC cells by exposing NPC cells to macrophage CM. Similar to macrophage CM treatment, γI-induced blockage of the intracellular cleavage of N-cadherin led to accumulated CTFs and reduced expression of MMP-9 after PMA treatment (Figs. 2 and 3). This result highlights the role of MMP-9 in N-cadherin cleavage and the signaling effect of N-cadherin cleavage in the upregulation of MMP-9 in inflammatory cells.

Carcinoma cells acquire their invasive phenotype by overexpressing various MMPs that cleave ECM components. MMPs regulate proliferation, adhesion, migration, and metastasis by cleaving cell-surface proteins [4]. MMP-inducedN-cadherin cleavage results in shedding of the extracellular NTF and generation of CTF1 (~40 kDa). CTF1 is further processed by a γ-secretase–dependent cleavage that releases the cytoplasmic domain (CTF2, ~35 kDa) [4]. PMA treatment decreased the expression of FL/N-cad (~130 kDa) (Fig. 2a, c). Treatment with γI induced accumulation of CTF1 (~40 kDa), which indicates a basal level of post-translational cleavage of N-cadherin in NPC cells under normal growth conditions (Fig. 2c). Notably, treatment with PMA or macrophage CM enhanced the γI-mediated CTF1 accumulation (Figs. 1 and 2), which indicates induced γ-secretase-dependent cleavage (or intracellular cleavage) of N-cadherin. Interestingly, macrophage CM induced N-cadherin cleavage without affecting E-cadherin cleavage in NPC cells (Fig. 1b). A similar result was shown in PMA-treated NPC cells (Fig. 2a), which suggests a unique role for N-cadherin cleavage in NPC cell invasion.

PMA may induce various MMPs to cleave the ECM. ADAM10, one of the many MMPs, has been extensively shown to cleave N-cadherin [9, 13]. However, we found that the expression of ADAM10 was not significantly affected in NPC cells in response to PMA treatment (Additional file 1: Figure S1). PMA time- and PKC-dependently induced the expression of MMP-9 in N-cadherin–presented NPC cell lines (Fig. 2). Whether the expression of MMP-9 contributes to the cleavage of N-cadherin remains for further study. Treatment with the pan-MMP inhibitor or specific MMP-9 inhibitor rescued the FL/N-cad level (Fig. 4a, b). Moreover, NTF/N-cad (~90 kDa) was present in the CM after PMA treatment (Fig. 8a), which indicates induced extracellular cleavage. The extracellular cleavage was further confirmed by treatment with activated recombinant MMP-9protein (Fig. 4c).

N-cadherin is one of the *trans-*membrane components of the adherens junction, and its expression and proteolytic cleavage might be closely associated with cancer cell invasion [13]. SiRNA knockdown of N-cadherin decreased PMA-mediatedMMP-9 expression (Fig. 6c). The recombinant activated MMP-9 alone could induce cleavage of N-cadherin (Fig. 4c) but not NPC cell invasion. The impact on cell invasion may depend on the intracellular cleavage of N-cadherin. Furthermore, PMA-enhancedMMP-9 protein level was efficiently suppressed by γI treatment and enhanced by ectopic expression of CTF2 (Figs. 3 and 9c). A recent study found that MMP-9 level in oral carcinoma cells was reduced to the normal level with lack of the cytoplasmic domain of N-cadherin, which suggests a vital role for CTFs of N-cadherin in regulating MMP-9 [8]. Consistently, our results indicated induced nuclear translocation of CTFs by PMA, which was suppressed by γI treatment. Moreover, the transactivation of MMP-9 was enhanced by ectopic expression of CTF2 in PMA-treated NPC cells (Fig. 9d). The results suggest a signaling role for CTFs/N-cad in modulating MMP-9 gene expression. The CTFs of N-cadherin form a complex with p120- and β-catenin and with PS1 at the plasma membrane (25). MMP-9 and MMP-12 cause N-cadherin shedding and induce vascular smooth muscle cell proliferation via β-catenin signaling [9]. We found that ectopic expression of CTF2 did not significantly induce MMP-9 expression (Fig. 9). The effect of CTF2 might be associated with the nuclear signaling of subcellular distribution. However, the mechanistic signaling of CTF2 needs to be investigated.

During tumor progression, a subset of primary tumor cells may undergo molecular changes, thereby leading to increased ability to survive, proliferate, invade, and even metastasize. Multiple signaling pathways control the expression of PMA-mediatedMMP-9 [27]. We found ERK1/2, p38, and NF-κB signaling pathways involved in PMA-inducedMMP-9 expression and promoter activity (Fig. 5). Treatment with MAPK inhibitors or NF-κBinhibitor suppressed the expression of PMA-mediatedMMP-9, which then suppressed the extracellular cleavage of N-cadherin and sequential intracellular cleavage of N-cadherin (Fig. 5). N-cadherin may have contributed to the invasive characteristics of carcinoma cells by upregulating MMP-9 in response to MAPK or NF-κB signaling, thereby increasing the aggressive metastasis. The effect of N-cadherin cleavage on NPC cell invasion might be a secondary inflammation response.

**Fig. 10:**
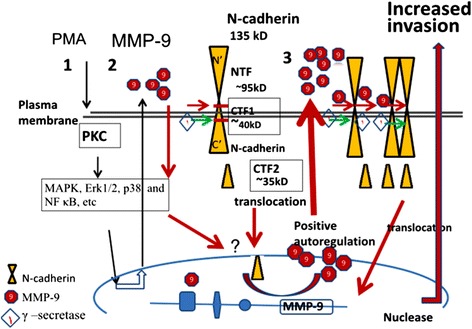
A model proposed for the cross-talk between N-cadherin cleavage and MMP-9 expression in NPC cell invasion

In addition, we found that MMP-9 in CM1 induced cell invasion and N-cadherin cleavage, which increased MMP-9 expression (Fig. 7c). However, the outside-in signaling effect of MMP-9 also depended on γ-secretase cleavage (Fig. 7d). This finding provided a possible link between MMP-9 and N-cadherin in inflammatory-mediated cell invasion. Given the restricted pattern of N-cadherin expression in carcinoma cells, the cleavage of N-cadherin enhancing cell invasion via increased MMP-9 levels indicated a cross-talk between MMP-9 and N-cadherin in carcinoma cells (Fig. 10). The impact of N-cadherin cleavage on MMP-9 expression might depend on the cellular context and be associated with level of intracellular cleavage.

## Conclusions

Elevated level of MMPs in the tumor microenvironment could activate stromal*-*like cells to drive cancer progression via further MMP production [29]. In this study, N-cadherin affected tumor progression by potentiating MMP-9 signaling in a cross-talk regulatory mechanism (Fig. 10). The expression of N-cadherin in metastatic cancer cells suggests that it may be a therapeutic target for advanced cancers. Our findings may reveal an important factor in diagnosing malignant diseases and designing effective anti-metastatic therapies.

## Additional file

Additional file 1: Figure S1. The expression of the precursor and active form of ADAM10 after PMA treatment. NPC cells were treated with PMA (100 nM) for the indicated times. Cell lysates underwent western blot analysis to detect levels of the precursor and active form of ADAM10. In response to PMA treatment, the expression of ADAM10 was not significantly changed in NPC cells. (TIF 645 kb)

## Abbreviations

BIM: Bisindolylmaleimide
CTF: C-terminal fragment
ECL: Enhanced Chemiluminescence
ECM: Extracellular matrix
EMT: Epithelial-to-mesenchymal transition
FL/N-cad: Full length N-cadherin
GM: GM6001
HRP: Horseradish peroxidase
MMP: Metalloproteinase
monoCM: Monocytes conditioned media
MP9I: MMP-9 inhibitor
mϕCM: Macrophage conditioned media
Ngi: Negative control siRNA
NPC: Human nasopharyngeal carcinoma
NTF: Amino-terminal fragment
PKC: Protein kinase C
PMA: Phorbol-12-myristate-13-acetate
QNZ: 6-Amino-4-(4-phenoxyphenylethylamino) quinazoline
SDS PAGE: Sodium dodecyl sulfate-polyacrylamide gel electrophoresis
siRNA: Small interfering RNAs
γI: L-685,458
