# Supplementary figures and images for: Interplay of N-Cadherin and matrix metalloproteinase 9 enhances human nasopharyngeal carcinoma cell invasion
Source: BMC Cancer. 2016 Oct 13;16:800. doi: 10.1186/s12885-016-2846-4 (PMC5064931; doi:10.1186/s12885-016-2846-4)

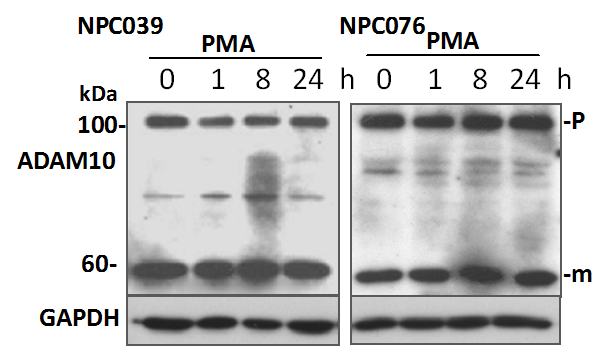

Supplement: Additional file 1: Figure S1. — The expression of the precursor and active form of ADAM10 after PMA treatment. NPC cells were treated with PMA (100 nM) for the indicated times. Cell lysates underwent western blot analysis to detect levels of the precursor and active form of ADAM10. In response to PMA treatment, the expression of ADAM10 was not significantly changed in NPC cells. (TIF 645 kb) [file 12885_2016_2846_MOESM1_ESM.tif]
